# Singular Death

**Published:** 1852-10

**Authors:** 


*Singular Death.*—In a late number of the Poughkeepsie Telegraph, is given an account of a most singular death. It appears that a young man, by the name of Webster, aged 27 years, had been suffering from excruciating tooth-ache for sometime; his face swelling, with accompanying signs of central sympathy, &c. He had a tooth extracted, but without relief, his troubles only increasing, until death put an end to all pain and disease.

It is strange that no full account of this case is to be had, although we believe there are dentists as well as physicians, resident of the town, and capable of contributing facts, derived from such a case, with precision and accuracy, involving as it does features of the most important character, and indispensable to improvement in practice. We almost daily see causes of complaint against the indolence and inability of our younger members, for failing to advance the results of their observation and practice. The above case, no doubt, is only one of many proofs of the necessity of a most complete and thorough knowledge of dental science, and perhaps one illustrating, if all the circumstances were known, the advantages to be derived from a medico-dental education, alike seviceable to both practitioners.

A similar case came under our observation in Nov. '51, but fortunately with a better result. The gentleman was a graduate of medicine, though not a practitioner; he had visited a dentist of some popular reputation, for the purpose of having a tooth filled, which had given him some uneasiness; a large cavity in its posterior approximal surface was hastily prepared and filled with amalgam, with the assurance of its skillful performance. The uneasiness, however, continued, and in spite of frequent visits and much temporising treatment, inflammation, with all its bad consequences, supervened. In the vain hope of restoring the tooth and surrounding parts to health, it was allowed to remain until the inflammation had extended to the whole alveolar border, and each tooth had become implicated in the disturbance; it was then extracted, but too late to produce any good effect; for the same day, delirium with a most severe and raging fever ensued. Dr. B. and M. of this city, were in attendance, and by their treatment the patient was restored to reason in a few days, and after an exposition of the cause of his illness, we were called in, and removed the bicuspids and molars of both sides, trusting to be able to retain the six front teeth; but with no success, as within three weeks it was found necessary to remove the remaining teeth; after which, the whole alveolar border exfoliated in small pieces at a time, occupying a space of four months. Considerable tumefaction existed over the palate and fauces, requiring frequent lancings, followed by the discharge of pus in greater or less quantities. The gums and mucous membrane healed very slowly, showing a constant disposition to ulcerate and slough. The bowels were kept open by saline cathartics, and astringent aromatic washes used, and a generous diet prescribed. Systematic exercise in pure air, with a constant care of every little minutia which are so commonly overlooked, finally restored the patient to good health again, when an upper set of teeth were inserted as nearly resembling his natural ones as possible.

